# In Vitro Evaluation of Spearmint Essential Oil (*Mentha spicata* L.) Supplementation on Gas Production, Rumen Fermentation, and Microbial Community Structure

**DOI:** 10.3390/ani16071007

**Published:** 2026-03-25

**Authors:** Chengzhen Huang, Jiamin Chen, Lin Wang, Lei Wang, Jiayi Li, Lifeng Dong

**Affiliations:** 1 Sino-US Joint Lab on Nutrition and Metabolism of Ruminant, Institute of Feed Research, Chinese Academy of Agricultural Sciences, Beijing 100081, China; hcz_0721@163.com (C.H.); 17631527522@163.com (J.L.); 2 College of Animal Science and Technology, Yangzhou University, Yangzhou 225009, China; yolo020529@163.com; 3 Joint International Research Laboratory of Agriculture and Agri-Product Safety, Institute of Agricultural Science and Technology Development, Yangzhou 225009, China; lynn1215@yzu.edu.cn; 4 Institute of Microbiology, Xinjiang Academy of Agricultural Sciences, Urumqi 830091, China; xjauwl@163.com

**Keywords:** spearmint essential oil, methane emission, rumen fermentation, microbial community, methyl-coenzyme M reductase

## Abstract

Reducing enteric methane emissions from ruminants has emerged as a critical environmental priority in the face of global climate change, given the substantial contribution of methane to agricultural greenhouse gas outputs. This study evaluated the potential of spearmint essential oil (SEO) to reduce methane production and enhance energy utilization efficiency using an in vitro rumen fermentation system. The experiment comprised a control (CON, no additive), three SEO doses (L-SEO: 100 mg/L; M-SEO: 200 mg/L; H-SEO: 400 mg/L), and a commercial essential oil blend (AGL: 150 mg/L). Results indicated that M-SEO and H-SEO significantly reduced methane production at 24 h from 58.11 mL/g DM in CON to 47.93 and 46.58 mL/g DM, respectively (*p* < 0.001), corresponding to reductions of 17.5% and 19.8%. Furthermore, M-SEO increased total volatile fatty acid concentration from 48.41 to 58.10 mmol/L and elevated the molar proportion of propionate, while significantly enhancing microbial crude protein production (*p* < 0.001). Microbial community analysis revealed that M-SEO increased bacterial alpha-diversity (Shannon index) (*p* = 0.001) and significantly enriched specific functional guilds, particularly the propionate-producing genus *Succiniclasticum* and the butyrate-producing genus *Butyrivibrio*. Interestingly, the abundance of dominant methanogens (*Methanobrevibacter*) was not reduced, suggesting a metabolic inhibition mechanism rather than a biocidal effect. Functional prediction analysis further supported this, indicating a downregulation of pathways associated with methanogenesis, including key enzymes such as methyl-coenzyme M reductase. In conclusion, SEO supplementation at 200 mg/L effectively reduced methane production by redirecting metabolic hydrogen toward propionate formation, without affecting overall fermentation. Therefore, the current study indicated that SEO could serve as a sustainable feed additive for mitigating enteric methane emissions in ruminants.

## Simple Summary

Extensive enteric methane mitigation approaches have been developed to alleviate anthropogenic greenhouse gas emissions and increase energy utilization efficiency in ruminant production. In this study, we investigated whether spearmint essential oil (SEO) could reduce methane production during rumen fermentation. Using an in vitro rumen fermentation system, different doses of SEO were evaluated. At a dose of 200 mg/L, SEO reduced methane production by 17.5%. The SEO supplementation increased total volatile fatty acids from 48.41 mmol/L in the control group to 58.10 mmol/L in the 200 mg/L SEO group, along with a higher molar proportion of propionate, which was associated with an increased abundance of propionate-producing bacteria (e.g., *Succiniclasticum*). In addition, the abundance of MCR was higher in the SEO supplemented group than in the control group. This study suggested that SEO can be a promising natural feed additive to mitigate enteric methane emission while improving the fermentation efficiency.

## 1. Introduction

Enteric methane from ruminant livestock has long been recognized as a major contributor to agricultural greenhouse gas emissions and a critical barrier to achieving climate-neutral food systems [1]. The global livestock sector accounts for approximately 15% of anthropogenic greenhouse gas emissions, with ruminants responsible for more than half of this total [2]. Methane (CH_4_), produced during enteric fermentation, possesses a global warming potential 28–34 times greater than that of CO_2_ over a 100-year horizon [3], and its relatively short atmospheric lifetime makes it a strategic target for near-term climate mitigation. Consequently, identifying effective nutritional or microbial strategies to suppress enteric methanogenesis has become a major research priority worldwide, particularly in regions where ruminant production plays a central role in agricultural emissions [4].

Reducing enteric CH_4_ emissions from ruminants has become a major focus of global efforts toward sustainable and climate-resilient livestock production. In recent years, several strategies have been explored to reduce enteric CH_4_ emissions from ruminants [5]. Among these, essential oils (EOs), which are plant-derived secondary metabolites rich in terpenes and phenolic compounds [6], have garnered increasing interest owing to their antimicrobial, antioxidant, and rumen-modulating properties [7]. The EOs can selectively inhibit methanogenic archaea while enhancing hydrogen utilization through the propionate formation pathway, thereby reducing CH_4_ emissions and maintaining rumen fermentation efficiency [8]. Several EO-based commercial formulations have already been developed, supported by growing evidence of their potential to decrease enteric CH_4_ and improve feed utilization [9,10]. In vitro studies demonstrated the potential of EOs to mitigate ruminal CH_4_ emissions through selective modulation of microbial fermentation. For example, oregano EO at 91 or 130 mg/L reduced total gas and CH_4_ production by enhancing the abundance of fiber-degrading bacteria and altering fermentation pathways [11]. Similarly, Benetel et al. [12] reported that ginger and thyme EOs at 50 mg/L achieved the greatest inhibition, reducing CH_4_ production by 13.69 and 10.62 mL/g, respectively. Additionally, Hart et al. [13] observed that supplementation with 1 g/d of a commercial EO blend over 22 weeks decreased CH_4_ emissions from approximately 438 to 411 g/d and increased milk yield from 28.3 to 31.2 kg/d, without altering milk composition. Silvestre et al. [14] also found that dietary inclusion of a capsicum–clove EO mixture (150–600 mg/head/day) linearly reduced CH_4_ emissions by up to 7.5%, while maintaining feed intake and lactation performance. Similar effects have also been reported in goats and sheep, where EOs such as eucalyptus oil and anise oil decreased CH_4_ production and modulated rumen microbiota structure [15].

Spearmint essential oil (SEO), derived from *Mentha spicata* L., is rich in monoterpenes such as carvone and limonene [16]. These compounds can alter microbial membrane permeability and disrupt energy metabolism, thereby inhibiting methanogenic archaea [17]. In vitro supplementation with 30 mg/g of limonene reduced CH_4_ production by approximately 34% [18], while carvacrol, another oxygenated monoterpene, was shown to suppress protein degradation and ammonia formation, thereby improving nitrogen utilization [19]. Moreover, blended EO formulations such as Agolin Ruminant have been widely studied for their practical application. Supplementation with 1 g/d of Agolin has been shown to reduce absolute CH_4_ output (L/d) and CH_4_ intensity (per kg of dry matter intake) without adverse effects on feed intake or milk yield. Meta-analysis results further indicate that long-term Agolin supplementation can lower CH_4_ emissions by about 10% and increase milk yield by approximately 3.6% [20]. Taken together, these findings highlight the promise of both single and mixed EO strategies for sustainable CH_4_ mitigation in ruminant nutrition.

Nevertheless, the specific mechanisms through which spearmint essential oil mitigates ruminal methanogenesis require further clarification. Specifically, its interactions with the rumen microbiome and the consequent effects on fermentation dynamics are not fully elucidated. However, the dose–response effects of SEO on CH_4_ yield, volatile fatty acid profiles, and the microbial community remain to be systematically delineated. Therefore, this study employs an in vitro fermentation system to investigate the influence of graded levels of SEO on these parameters. The objective is to identify the optimal inclusion level that effectively reduces CH_4_ emissions while maintaining or improving ruminal fermentation efficiency, thereby assessing SEO’s potential as a natural strategy for sustainable livestock production.

## 2. Materials and Methods

### 2.1. Experimental Materials and Design

Rumen fluid was collected from three non-lactating Holstein dairy cows fitted with permanent ruminal cannulas, with a body weight of (650 ± 30) kg. All experimental procedures involving animals were approved by the Animal Welfare Committee of Yangzhou University (Approval No. 202503758). Spearmint essential oil was provided by DadHank (Chengdu) Biotech Corp. (Chengdu, China), with the main components being carvone (60%) and limonene (18%). A stock solution was prepared by dissolving the spearmint oil in ethanol, with the final ethanol concentration maintained below 0.5% [21,22]. The fermentation substrate was a total mixed ration (TMR) with a forage-to-concentrate ratio of 6:4, whose composition and nutritional levels are shown in Table 1.

Referring to the method of Menke et al. [24], the following solutions were prepared for immediate use: Solution A (0.1 L) containing 13.2 g CaCl_2_·2H_2_O, 10.0 g MnCl_2_·4H_2_O, 1.0 g CoCl_2_·6H_2_O, and 8.0 g FeCl_3_·6H_2_O; Solution B (1 L) containing 4.0 g NH_4_HCO_3_ and 35.0 g NaHCO_3_; Solution C (1 L) containing 5.7 g Na_2_HPO_4_, 6.2 g KH_2_PO_4_, and 0.6 g MgSO_4_·7H_2_O; a reducing agent solution (100 mL) containing 0.16 g NaOH and 0.625 g Na_2_S·9H_2_O; and a 0.1% resazurin indicator solution (Solution D). The rumen buffer was prepared by mixing 400 mL of distilled water, 0.1 mL of Solution A, 200 mL of Solution B, 200 mL of Solution C, 1 mL of resazurin solution, and 40 mL of reducing agent solution.

A total of six treatment groups were established. These included a negative control group (CON, containing substrate without spearmint essential oil), three spearmint essential oil treatment groups (L-SEO, M-SEO, and H-SEO, with final concentrations of 100, 200, and 400 mg/L, respectively), a positive control group (AGL, with 150 mg/L of Agolin), and a substrate-free blank group (Blank, containing only buffered rumen fluid without substrate or additives) used for correcting gas production values. Each treatment was performed in four replicates. Separate sets of fermentation bottles were prepared for each sampling time point (6, 24, and 48 h). At each designated time point, the corresponding bottles were removed and destructively sampled for gas composition analysis.

### 2.2. In Vitro Rumen Fermentation Procedure

Rumen fluid was collected from various sites in the ventral sac of the rumen before morning feeding. The collected fluid was mixed thoroughly and filtered through four layers of sterile gauze. The filtered rumen fluid was then combined with pre-warmed (39 °C) rumen buffer in a 1:2 ratio to prepare the rumen fermentation medium.

The fermentation system consisted of 180 mL anaerobic fermentation bottles. Each bottle was charged with 500 mg of substrate and 75 mL of the culture medium, which was a 1:2 mixture of rumen fluid and artificial buffer. Different concentrations of spearmint essential oil were added to the bottles according to the experimental groups. The bottles were continuously purged with CO_2_ for 30 s before being sealed with butyl rubber stoppers. They were then placed in a constant temperature oscillator (Model THZ-320, Shanghai Jinghong Experimental Equipment Co., Ltd., Shanghai, China) and incubated at 39 °C and 80 r/min for 6, 24, or 48 h, depending on the designated sampling time point.

### 2.3. Determination of Gas Production

Gas pressure in each fermentation bottle was measured at 2, 4, 6, 8, 10, 12, 24 h, and 48 h using a digital pressure sensor (Model DPG1000B15PSIG-5, Cecomp Electronics, Libertyville, IL, USA). The gas production volume was calculated according to the formula described by Datsomor et al. [25]:

(1) $$V_{gas}=V_{j}\times P_{psi}\times 0.068004084,$$

where $V_{gas}$ is the gas volume (mL) at 39 °C, $V_{j}$ is the headspace volume from the fermentation broth surface to the top of the bottle (mL), and $P_{psi}$ is the bottle pressure (psi). The calculated gas production was corrected using values obtained from blank cultures.

Gas samples (5 mL) were collected from separate fermentation bottles that were independently incubated and destructively sampled at 6, 24, and 48 h, respectively. Samples were stored in gas sampling bags and analyzed for gas composition using a gas chromatograph (GC9800, Shanghai Techcomp Chromatograph Co., Ltd., Shanghai, China). The analytical conditions were as follows: column temperature at 110 °C, TCD detector temperature at 200 °C, bridge current at 50 mA, and argon as the carrier gas.

### 2.4. Determination of Rumen Fermentation Parameters

At the end of fermentation, the fermentation bottles were immediately placed in an ice bath to terminate the process. The pH value was measured using a pH meter (PHS-3C, Shanghai Puchun Measuring Instrument Co., Ltd., Shanghai, China). Subsequently, 24 h fermentation broth was collected and stored at −20 °C for subsequent determination of volatile fatty acids (VFA), ammonia nitrogen (NH_3_-N), and microbial crude protein (MCP). The residue in the bottles was washed with sterile distilled water, centrifuged, and then used for determining the dry matter degradation rate. The residue was dried at 65 °C for 48 h, weighed, and the dry matter degradation rate was calculated.

For VFA analysis, the fermentation broth was centrifuged at 12,000 r/min for 10 min. One milliliter of the supernatant was mixed with 0.2 mL of meta-phosphoric acid (20%, containing 60 mmol/L crotonic acid) and stored at −20 °C overnight. After thawing, the mixture was centrifuged again at 12,000 r/min for 10 min (4 °C). The supernatant was passed through a 0.22 μm aqueous phase filter, and 1 μL of the filtrate was injected into a gas chromatograph (GC-9800, Shanghai Techcomp Chromatograph Co., Ltd.) for VFA quantification. Separation was performed using a capillary column (30.00 m × 0.32 mm × 0.25 μm, Agilent Technologies, Beijing, China) with the following conditions: injector temperature 220 °C, initial column temperature 80 °C (programmed to increase to 125 °C at 3 °C/min), FID detector temperature 220 °C, nitrogen as the carrier gas, and crotonic acid as the internal standard.

The reaction of alkaline sodium hypochlorite with phenol was quantitatively analyzed to determine NH_3_-N as described in Broderick and Kang [26]. The concentration of MCP was detected by the BCA rapid protein quantitative kit (Nanjing Jiancheng, Nanjing, China).

### 2.5. Metagenomic Sequencing and Analysis

At the end of the in vitro rumen fermentation, 5 mL of fermentation fluid was collected from each treatment bottle (six treatments with four replicates per treatment). After sample collection, they were immediately stored in a −80 °C ultra-low-temperature freezer for future use. The DNA extraction was performed using the E.Z.N.A.^®^ soil DNA kit (Omega Bio-tek, Norcross, GA, USA). After extraction, purity is detected using 1% agarose gel electrophoresis and a NanoDrop2000 spectrophotometer (Thermo Fisher Scientific, Shanghai, China) (A260/A280 > 1.8, A260/A230 > 2.0); any unqualified samples are re-extracted. Sequencing of the 16S rRNA gene and metagenomic raw data acquisition were performed by Anshan Biotechnology Co., Ltd. (Tianjin, China).

Raw metagenomic reads were processed with FASTP (version 0.12.4) [27] for quality control, and high-quality sequences were assembled at different sequencing depths using MEGAHIT (version 1.1.3) (https://github.com/voutcn/megahit, accessed on 18 August 2025). Open reading frame (ORF) prediction was conducted with Prodigal, and genes with nucleotide lengths ≥ 1000 bp were translated into amino acid sequences. The predicted gene sequences from all samples were clustered with CD-HIT (version 4.8.1) (https://www.bioinformatics.org/cd-hit/, accessed on 18 August 2025) using thresholds of 95% identity and 90% coverage. The longest sequence in each cluster was retained as the representative sequence to construct a non-redundant gene catalog.

Functional annotation of the non-redundant gene set was performed through BLAST (version 2.14.0) searches (e-value ≤ 1 × 10^−5^) against the NCBI NR and KEGG GENES databases, with particular focus on genes related to nitrogen metabolism. Co-occurrence network analysis was conducted using the microeco package (version 1.10.0) in R (version 4.5.2), and network visualization was performed with Gephi (version 0.10.1).

### 2.6. Statistical Analysis

All statistical analyses were performed using R software (version 4.5.2, R Core Team). Data were checked for normality using the Shapiro–Wilk test prior to analysis.

Total gas production and gas composition variables (methane, carbon dioxide, and hydrogen production) were analyzed using a general linear model based on two-way analysis of variance (ANOVA). Treatment, incubation time, and their interaction were included as fixed effects. Since separate fermentation bottles were independently prepared and destructively sampled at each time point (6, 24, and 48 h), the observations across different time points were derived from independent experimental units, thereby satisfying the independence assumption of the two-way ANOVA model. The statistical model was defined as:

(2) $$Y_{ij}=\mu +T_{i}+{Time}_{j}+{\left(T\times Time\right)}_{ij}+\varepsilon_{ij},$$

where $Y_{ij}$ represents the observed gas production variable, μ is the overall mean, $T_{i}$ is the fixed effect of treatment, ${Time}_{j}$ is the fixed effect of incubation time, ${\left(T\times Time\right)}_{ij}$ is the interaction between treatment and time, and $\varepsilon_{ij}$ is the residual error. When significant effects were detected, post hoc comparisons were conducted using Tukey-adjusted multiple comparisons.

Fermentation parameters and alpha diversity indices of the rumen microbial community were analyzed using one-way ANOVA followed by Tukey’s honestly significant difference (HSD) test when the data met the assumptions of normality and homogeneity of variance. For data that did not conform to a normal distribution, the Kruskal–Wallis test was applied, and multiple comparisons were conducted using Dunn’s post hoc test with false discovery rate (FDR) correction.

Beta diversity was assessed based on Bray–Curtis dissimilarity matrices and visualized by principal coordinate analysis (PCoA). Differences in microbial community structure among treatments were evaluated using permutational multivariate analysis of variance (PERMANOVA) with 999 permutations. Differential analysis of microbial taxa at the genus and species levels, as well as functional pathways including KEGG orthologs, metabolic pathways, and carbohydrate-active enzymes (CAZymes), was conducted using the Kruskal–Wallis test followed by Dunn’s post hoc test. Statistical significance was declared at *p* < 0.05.

## 3. Results

### 3.1. Effect of Spearmint Essential Oil and Agolin on Gas Production and Composition

**Figure 1:**
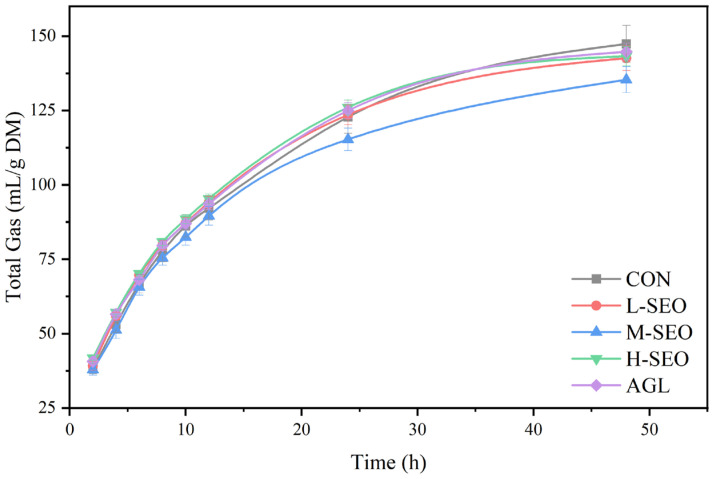
Cumulative gas production during 48 h of in vitro rumen fermentation with different additives. CON = without any additives; L-SEO, M-SEO, H-SEO = experimental diet plus 100, 200, and 400 mg/L spearmint essential oil, respectively; AGL = experimental diet plus 150 mg/L Agolin.

The dynamic profiles of in vitro gas production over the 48h incubation period are illustrated in Figure 1. Generally, all treatment groups exhibited a typical asymptotic fermentation curve, characterized by a rapid increase in gas volume during the initial 10 h followed by a gradual deceleration as the substrate was depleted. The supplementation of SEO resulted in a downward shift in the gas production curves relative to the control. Specifically, the M-SEO lines showed a distinct separation from the CON group, indicating a suppression of fermentation extent.

As shown in Table 2, temporal dynamics were observed in gas production. No significant differences in total gas production or individual gas components were found among treatments at 6 h (*p* > 0.05). However, at 24 h, total gas production in the H-SEO group was significantly lower than in the CON and L-SEO groups (*p* < 0.001). While the M-SEO group showed no significant difference in total gas production compared with CON at 24 h, both M-SEO and H-SEO treatments significantly reduced CH_4_ production relative to CON at this time point (*p* < 0.05). Notably, at 48 h, the inhibitory effect of H-SEO on CH_4_ production diminished, showing no significant difference from CON (*p* > 0.05), whereas the M-SEO group sustained a significantly lower CH_4_ yield compared with CON (*p* < 0.05). Regarding carbon dioxide (CO_2_), production at 24 h was significantly decreased in the H-SEO group compared with CON (*p* < 0.05), while M-SEO showed no significant difference. No significant differences were detected in total gas, CH_4_, or CO_2_ production between H-SEO and AGL at 24 h. Hydrogen production did not differ significantly among groups at any time point (*p* > 0.05).

### 3.2. Effect of Spearmint Essential Oil and Agolin on Fermentation Parameters

As shown in Table 3, the rumen fermentation parameters at 24 h were significantly altered by the additives. Regarding pH, values ranged between 6.43 and 6.47 (*p* = 0.004). Specifically, the pH values in the H-SEO and AGL groups were significantly higher than that in the CON group (*p* < 0.05), whereas the L-SEO and M-SEO groups showed no significant differences compared with CON. In terms of nitrogen metabolism, the NH_3_-N concentration was significantly lower in the H-SEO and AGL groups compared with CON (*p* < 0.05), with no significant differences observed between the L-SEO or M-SEO groups and the CON. Conversely, MCP synthesis was significantly enhanced in the M-SEO, H-SEO, and AGL groups compared with CON (*p* < 0.05). However, the MCP concentration in the L-SEO group did not differ significantly from the control (*p* > 0.05).

Total VFA concentration was significantly influenced by the treatments (*p* = 0.042), with the M-SEO group achieving the highest concentration (58.10 mmol/L). For individual fatty acids, significant increases were observed in propionate, butyrate, and isovalerate in the M-SEO group compared with CON (*p* < 0.05). Specifically, the propionate concentration in M-SEO was significantly higher than in the CON, L-SEO, and AGL groups, although it did not differ statistically from the H-SEO group. Similarly, butyrate and isovalerate concentrations in the M-SEO group were significantly higher than those in the CON, AGL, and L-SEO groups (*p* < 0.05), whereas they did not differ statistically from the H-SEO group. The caproate concentration in both M-SEO and H-SEO groups was significantly higher than that in the CON and AGL groups (*p* = 0.005). No significant differences were detected in the concentrations of acetate, isobutyrate, or valerate among the groups (*p* > 0.05). The acetate-to-propionate (A/P) ratio showed a decrease in the M-SEO group (4.38) compared with CON (4.90), but this difference was not statistically significant (*p* = 0.285).

Additionally, the additives had no significant effect on in vitro dry matter digestibility (IVDMD), which ranged from 53.82% to 58.05% across all groups (*p* = 0.629).

### 3.3. Effect of Spearmint Essential Oil and Agolin on Microbial Diversity and Composition

The alpha diversity indices for bacterial and archaeal communities in the 24 h fermentation fluid were presented in Table 4. For the bacterial community, the richness indices (Chao1 and ACE) showed no significant differences among treatments (*p* > 0.05). There seemed to be a decrease in the Chao 1 for the SEO dose, but it was not significantly different (H-SEO vs. CON: *p* = 0.208). However, community diversity and evenness were significantly altered by the medium dose. Specifically, the M-SEO group exhibited a significantly higher Shannon index (8.96) and Simpson index (0.989) compared with the CON group (*p* < 0.05). Regarding the archaeal community, the overall alpha diversity remained relatively stable across most indices (*p* > 0.05 for Simpson and Pielou). However, the Shannon index was significantly elevated in the M-SEO group compared with the CON (8.115 vs. 8.077; *p* < 0.05), mirroring the trend observed in the bacterial community.

**Figure 2:**
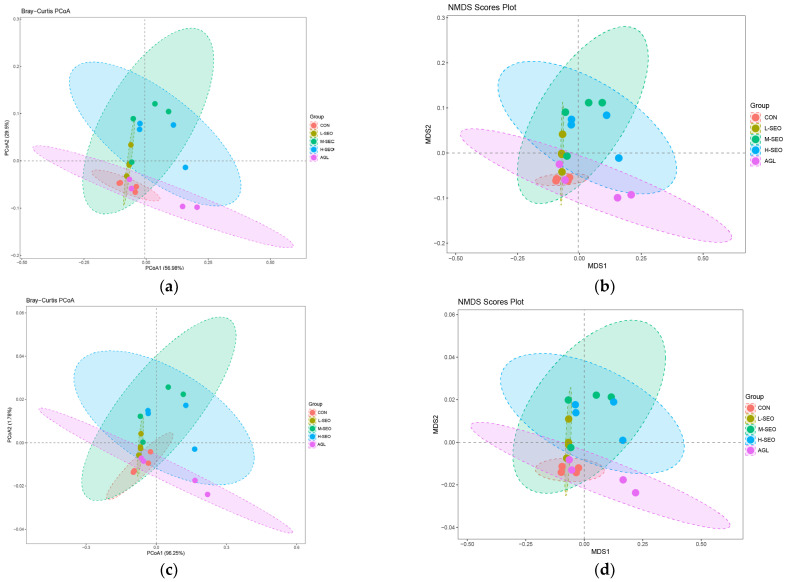
(**a**) Principal coordinate analysis (PCoA) and (**b**) nonmetric multidimensional scaling (NMDS) of the rumen bacterial community based on Bray–Curtis distances separating the control and essential oil supplementation groups (spearmint essential oil and Agolin). (**c**) Principal coordinate analysis (PCoA) and (**d**) NMDS of the rumen archaea community based on Bray–Curtis distances separating the CON and essential oil supplementation groups. CON = without any additives; L-SEO, M-SEO, H-SEO = experimental diet plus 100, 200, and 400 mg/L spearmint essential oil, respectively; AGL = experimental diet plus 150 mg/L Agolin.

Beta diversity analysis based on PCoA and NMDS revealed distinct clustering patterns for the bacterial community (Figure 2). PERMANOVA analysis confirmed that the bacterial community structure was significantly influenced by the treatments (R^2^ = 0.47, *p* = 0.009). Pairwise comparisons indicated that the CON group significantly differed from the L-SEO, M-SEO, and H-SEO groups (*p* < 0.05 for all), whereas no significant difference was observed between the CON and AGL groups (*p* = 0.166). In contrast, regarding the archaeal community, although the PCoA plot displayed some visual separation, the PERMANOVA results indicated no statistically significant differences in community structure among the groups (R^2^ = 0.34, *p* = 0.152).

**Figure 3:**
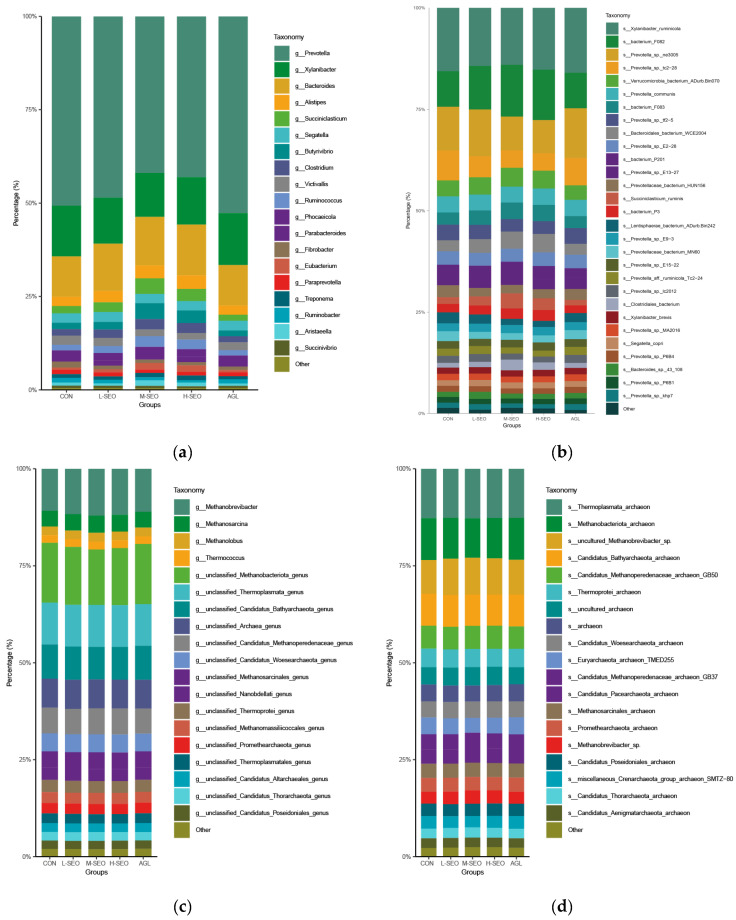
Comparative analysis of microbial community structure between the control and essential oil supplementation groups (spearmint essential oil and Agolin). Stacked bar plots depict the relative abundance of (**a**) bacterial genera, (**b**) bacterial species, (**c**) archaeal genera, and (**d**) archaeal species. CON = without any additives; L-SEO, M-SEO, H-SEO = experimental diet plus 100, 200, and 400 mg/L spearmint essential oil, respectively; AGL = experimental diet plus 150 mg/L Agolin.

At the genus level (Figure 3a), the bacterial community was predominantly composed of *Prevotella*, followed by *Xylanibacter* and *Bacteroides*, constituting the core microbiome across all treatments. The abundance of *Succiniclasticum*, a key propionate-producing genus, was significantly higher in the M-SEO and H-SEO groups compared with the control (*p* < 0.05). Similarly, *Butyrivibrio* and *Ruminococcus*, genera associated with butyrate production and fiber degradation, were significantly elevated in the M-SEO and H-SEO groups relative to the control (*p* < 0.05). The AGL group showed a numerically higher abundance of *Xylanibacter* and several *Prevotella* species, though these differences did not reach statistical significance. At the species level (Figure 3b), *Succiniclasticum ruminis* showed a consistent enrichment in the M-SEO and H-SEO groups relative to the CON (*p* < 0.05). Among *Prevotella*-affiliated species, *Prevotella* sp. tc2-28 and *Prevotella* sp. tf2-5 were significantly higher in the AGL group compared with the L-SEO, M-SEO, and H-SEO groups (*p* < 0.05). Regarding the archaeal community (Figure 3c,d), *Methanobrevibacter* and *Thermoplasmata* archaea were the dominant taxa across all groups. The abundance of *Methanobrevibacter* and *uncultured Methanobrevibacter* sp. showed a numerical increase in the M-SEO, H-SEO, and AGL groups. A similar pattern was observed for *Methanosarcina* and *Methanobacteriota* archaea.

### 3.4. Correlation Analysis and Functional Prediction

**Figure 4:**
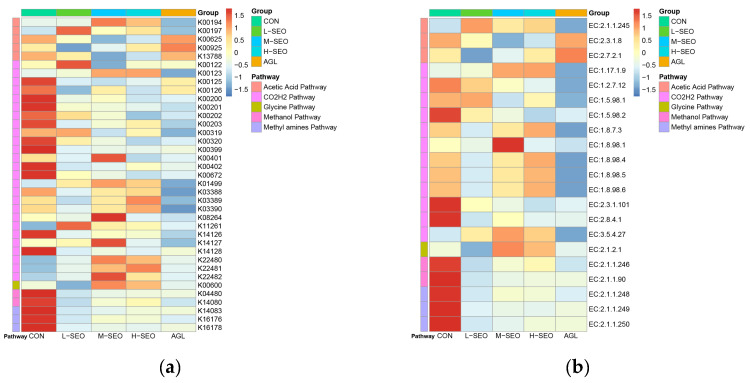
(**a**) Heatmap depicting the relative abundance of key genes involved in methanogenic pathways between the control and essential oil supplementation groups (spearmint essential oil and Agolin). (**b**) Heatmap depicting the relative abundance of key methanogenic enzymes in methanogenic pathways between the control and essential oil supplementation groups. CON = without any additives; L-SEO, M-SEO, H-SEO = experimental diet plus 100, 200, and 400 mg/L spearmint essential oil, respectively; AGL = experimental diet plus 150 mg/L Agolin.

The relative abundance of key genes involved in methanogenic pathways is shown in Figure 4a. Heatmap visualization revealed a clear alteration in methanogenic gene profiles in response to essential oil supplementation compared with the control group. Overall, the CON exhibited higher relative abundances of methanogenesis-related genes. In contrast, all essential oil-supplemented groups showed varying degrees of reduction in gene abundance across multiple pathways. Among the essential oil treatments, suppression patterns were gene-specific and non-linear with dose. The L-SEO showed moderate reductions in several genes, whereas more pronounced decreases were detected in the M-SEO and H-SEO for certain pathways. The H-SEO group displayed the stronger suppression in genes associated with the CO_2_/H_2_ reduction pathway (e.g., K00125) and the acetic acid pathway (e.g., K00625, K13788). Genes involved in the methanol and methylamine pathways (e.g., K00399, K00402) were also affected, with a general downward trend across treatments. The AGL exhibited a suppression pattern similar to that observed in the M-SEO group. The relative abundance of key methanogenic enzymes is presented in Figure 4b. Consistent with the gene-level results, essential oil supplementation resulted in an overall decrease in the abundance of enzymes involved in methanogenic pathways. Several core enzymes, including those associated with the final steps of methane formation (e.g., EC:2.8.4.1, EC:1.8.98.1), showed lower relative abundances in the essential oil-treated groups compared with the control. This reduction was particularly evident in the M-SEO and H-SEO groups for hydrogenotrophic enzymes (e.g., EC:2.3.1.101), with stronger suppression in methylotrophic enzymes (e.g., EC:2.1.1.90, EC:2.1.1.249, EC:2.1.1.250) across SEO doses. The L-SEO group exhibited milder changes in some enzymes, while the Agolin treatment produced enzyme abundance patterns comparable to those observed in the medium-dose spearmint essential oil group, indicating similar impacts on methanogenic metabolic functions.

**Figure 5:**
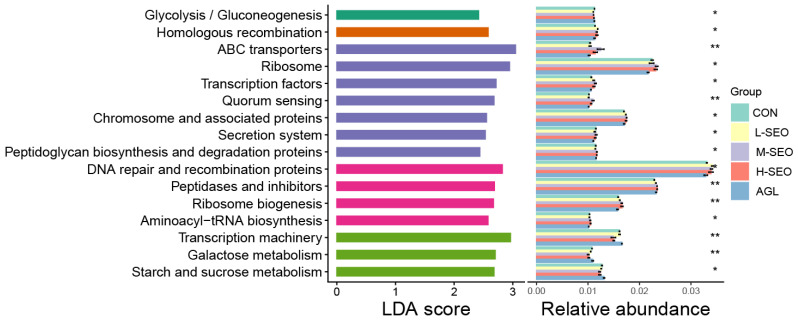
Functional enrichment analysis of metabolic and cellular processes between the control and essential oil supplementation groups (spearmint essential oil and Agolin). CON = without any additives; L-SEO, M-SEO, H-SEO = experimental diet plus 100, 200, and 400 mg/L spearmint essential oil, respectively; AGL = experimental diet plus 150 mg/L Agolin. * *p* < 0.05; ** *p* < 0.01.

The LEfSe analysis identified differentially abundant functional pathways across treatment groups (Figure 5). Carbohydrate metabolism pathways (glycolysis/gluconeogenesis, galactose metabolism, starch and sucrose metabolism) showed higher enrichment in the control group. In contrast, DNA repair and recombination proteins, homologous recombination, and chromosome-associated proteins were significantly enriched in the M-SEO and H-SEO groups, indicating a stress response to essential oil exposure. Protein synthesis pathways (ribosome, aminoacyl-tRNA biosynthesis, transcription machinery) and peptidases and inhibitors displayed differential abundance, with notable enrichment in the M-SEO group. The ABC transporters and secretion systems also varied across groups, suggesting altered cellular transport mechanisms. The L-SEO group showed intermediate profiles, while the Agolin group resembled M-SEO in functional enrichment patterns.

## 4. Discussion

### 4.1. Effect of Spearmint Essential Oil on Gas Production and Composition

In this study, SEO demonstrated a clear dose-dependent modulation of gas production, particularly evident at 24 h. The CH_4_ yield in the H-SEO group dropped from 58.11 to 46.58 mL/g DM, a 19.8% reduction compared with the control. While H-SEO exhibited strong inhibition initially, its efficacy on CH_4_ mitigation diminished by 48 h, suggesting a potential adaptation of the microbial community or a recovery of methanogenic activity at high doses. In contrast, the M-SEO treatment achieved a 17.5% reduction in CH_4_ at 24 h. Notably, it sustained this suppression at 48 h without significantly compromising total gas production at either time point. These findings indicate a threshold response where M-SEO offers a more sustainable balance between CH_4_ mitigation and fermentation efficiency than H-SEO, which may overly inhibit general fermentation.

Comparable reductions have been reported for other essential oils, such as clove, oregano, eucalyptus, and thyme, which decreased CH_4_ by 15–30% in vitro [28,29,30]. The magnitude observed in our study aligns with these findings [31]. Notably, the AGL group reduced CH_4_ by 13.4% at 24 h (58.11 to 50.31 mL/g DM) and total gas by 11.4% (151.69 to 134.37 mL/g DM). This CH_4_ reduction is slightly higher than the average 8.8% reported by [20], suggesting that Agolin’s efficacy may vary with diet composition and experimental conditions [32], and that spearmint oil (particularly M-SEO) can achieve comparable or even stronger, more persistent effects under similar in vitro conditions.

Carbon dioxide output decreased significantly from 74.99 to 56.42 mL/g DM under H-SEO at 24 h, a reduction of approximately 24.8%, indicating partial suppression of overall fermentation activity. Similar patterns have been observed when high doses of essential oils broadly inhibit carbohydrate degradation [9,33]. Hydrogen production remained low and was statistically unaffected by treatments at all time points (*p* > 0.05). For instance, at 24 h, hydrogen production in the AGL group was similar to the control (0.76 vs. 0.77 mL/g DM), indicating that the inhibition of methanogenesis did not lead to significant hydrogen accumulation in the headspace, possibly due to the redirection of metabolic hydrogen into alternative sinks such as propionate [34,35].

Overall, the present findings confirm that essential oils can modulate gas production in a dose- and time-dependent manner. At moderate concentrations, SEO effectively and persistently suppresses CH_4_ without markedly reducing total gas, indicating a favorable CH_4_ mitigation–fermentation balance.

### 4.2. Effect of Spearmint Essential Oil on Fermentation Parameters

In this study, SEO supplementation significantly improved nitrogen utilization efficiency, evidenced by the marked increase in MCP synthesis. The commercial blend AGL achieved a comparable enhancement. This enhancement in microbial protein synthesis, concurrent with the reduction in NH_3_-N observed in the SEO and AGL groups, suggests a more efficient conversion of dietary nitrogen into microbial biomass. This aligns with the hypothesis that essential oils inhibit hyper-ammonia-producing bacteria (HAP), such as *Clostridium sticklandii* and *Peptostreptococcus anaerobius*, thereby reducing distinct deamination and favoring nitrogen incorporation into microbial protein [36,37]. Similar improvements in MCP synthesis following essential oil supplementation were previously noted by Tekippe et al. [38] and Thao et al. [39].

The inclusion of M-SEO, in particular, exerted a stimulatory effect on rumen fermentation profiles. Total VFA concentration in the M-SEO group increased by approximately 20% compared with the control (58.10 vs. 48.41 mmol/L), indicating enhanced overall microbial activity. Notably, propionate concentration rose significantly from 7.13 to 9.21 mmol/L (+29%) in the M-SEO group. This shift suggests a redirection of carbon flow towards glucogenic pathways, which serves as a competitive hydrogen sink, thereby contributing to the methane reduction observed in this study [11]. Regarding the acetate-to-propionate ratio, although a numerical decrease was observed in the M-SEO group (4.38) compared with the control (4.90), this difference was not statistically significant (*p* = 0.285). Therefore, the fermentation shift described here is primarily characterized by the absolute increase in propionate production rather than a significant alteration in the ratio itself.

It is worth noting that M-SEO supplementation also significantly increased the concentrations of butyrate and isovalerate (*p* < 0.05), unlike the other treatment groups. Isovalerate acts as a critical growth factor for cellulolytic bacteria (e.g., *Ruminococcus albus*) [40], which may explain why dry matter digestibility was maintained despite the shifts in fermentation. Furthermore, a marked increase in caproate concentration was observed, surging from 0.06 mmol/L in the control to 0.29 mmol/L in the M-SEO treatment—a nearly fivefold enhancement. This response suggests a selective stimulation of chain-elongation pathways [41], likely driven by the interaction of essential oil metabolites with specific functional guilds such as *Clostridium* species [37].

When comparing the strategies, the AGL group exhibited MCP levels similar to those of the H-SEO treatment, yet its total VFA concentration (49.23 mmol/L) remained statistically comparable to the control. This indicates that AGL enhances microbial protein synthesis while maintaining a more conservative fermentation profile. A similar trend was reported in a meta-analysis by Belanche et al. [20], noting that commercial blends often exert moderate effects on total fermentation activity. In contrast, the M-SEO appears to offer a dual benefit: it improved microbial protein synthesis by nearly 30% while simultaneously stimulating VFA production (specifically propionate and butyrate). Overall, these findings suggest that while both SEO and AGL beneficially modulate rumen fermentation, M-SEO provides a more robust stimulation of fermentation activity, optimizing the trade-off between methane mitigation and nutrient availability.

### 4.3. Effect of Spearmint Essential Oil on Microbial Diversity and Composition

The rumen microbial community data offer critical insights into the ecological mechanisms underlying the fermentation shifts observed in this study. The SEO administration enhanced bacterial alpha diversity, specifically increasing the Shannon and Simpson indices in the M-SEO group. This suggests that moderate SEO supplementation promotes a more even distribution of bacterial taxa, avoiding the dominance of a single species. Beta diversity analysis (PCoA, NMDS) further revealed that the M-SEO group formed a distinct cluster separate from the control, indicating a significant structural reorganization of the bacteriome [42].

At the genus and species levels, the specific enrichment of functional guilds provides a biological explanation for the altered VFA profiles (Table 3). A pivotal finding of this study was the significant enrichment of *Succiniclasticum* (specifically *S. ruminis*) in the M-SEO and H-SEO groups. *Succiniclasticum* specializes in converting succinate to propionate, a pathway that serves as a competitive hydrogen sink [43]. The proliferation of this genus aligns perfectly with the increased propionate proportions observed in our fermentation results. This suggests that SEO redirects metabolic hydrogen away from methanogenesis and towards propionate synthesis by selectively stimulating succinate-utilizing bacteria.

Furthermore, contrary to some reports indicating that essential oils inhibit fibrolytic and butyrate-producing bacteria [44], our data showed a substantial increase in *Butyrivibrio* and *Ruminococcus* in both the M-SEO and H-SEO group. *Butyrivibrio* is a primary producer of butyrate, and its enrichment explains the elevated butyrate concentrations observed with SEO supplementation. However, consistent with the dose-dependent nature of essential oils [45], the stimulatory effect on these *Firmicutes* members appeared most pronounced at the M-SEO group, suggesting that higher concentrations of SEO might limit further population expansion or total VFA production.

The commercial blend Agolin induced a microbial shift distinct from SEO. While M-SEO favored *Succiniclasticum* and *Butyrivibrio*, the AGL group was characterized by the specific enrichment of several *Prevotella* species (e.g., *Prevotella* sp. tc2-28 and tf2-5), alongside a numerically higher abundance of *Xylanibacter*. This indicates that Agolin may primarily modulate fermentation by enhancing the population of xylanolytic bacteria, thereby supporting fiber degradation [46]. This observation is consistent with in vivo trials reporting that Agolin alters rumen microbial structure to improve feed efficiency without reducing taxonomic diversity [20,47].

Perhaps the most intriguing finding was the decoupling of methane production from methanogen abundance. Despite the significant reduction in methane production in the SEO and AGL groups (Table 2), the relative abundance of dominant methanogens, including *Methanobrevibacter* and *Thermoplasmata* archaeon, did not decrease; in fact, they showed a numerical increase. This challenges the assumption that essential oils mitigate methane simply by reducing the population of methanogens. Instead, these results strongly support the hypothesis that spearmint essential oil functions by inhibiting the metabolic activity or specific enzymatic pathways of methanogens rather than via a direct biocidal effect [48]. Essential oils may inhibit key enzymes such as methyl-coenzyme M reductase (MCR) or disrupt the electron transport system within archaeal cells without causing cell lysis [31,49]. Consequently, the methanogen community remains structurally intact but metabolically less active in terms of CH_4_ synthesis. This “metabolic inhibition” mechanism, combined with the “hydrogen redirection” driven by the enriched *Succiniclasticum* population, represents the dual mode of action by which SEO mitigates methane emissions.

### 4.4. Correlation Analysis and Functional Prediction

The functional analysis in the present study suggests that SEO mitigates methanogenesis primarily through the orchestrated downregulation of core methanogenic enzymatic machinery. Specifically, the MCR and heterodisulfide reductase (HDR) emerged as the central inhibition targets. The MCR is the prerequisite enzyme complex catalyzing the terminal, rate-limiting step of CH_4_ formation in all methanogenic pathways [50,51,52], while HDR is critical for maintaining archaeal redox balance by regenerating the reduced coenzymes essential for electron flow toward MCR [53,54].

Our metagenomic profiles revealed a non-linear, dose-specific suppression in both MCR- and HDR-associated gene abundances, with overall lower relative abundances observed across the SEO-treated groups compared with the control. Notably, the inhibitory profile of the M-SEO group paralleled that of the commercial additive Agolin. This molecular suppression corresponds with the observed ~20% reduction in CH_4_ production, supporting the hypothesis that SEO-induced impairment of HDR restricts coenzyme regeneration. This blockage likely disrupts the electron transfer chain, subsequently limiting MCR catalytic efficiency [55]. These findings align with previous reports where phytogenic additives or essential oils suppressed archaeal activity and methane production by downregulating methanogenic gene expression [56,57].

The observed enzymatic suppression is likely attributable to the bioactive monoterpenoids in spearmint oil, such as carvone and limonene. These lipophilic compounds are known to disrupt archaeal cell membranes, dissipate proton gradients, and interfere with membrane-bound electron-transfer complexes [58,59]. The concomitant downregulation of the Fe-S-cluster enzyme HDR and the MCR complex [60] in our study is consistent with the mode of action described in prior in vitro and in vivo studies, which posit that essential oils compromise the transcription or stability of methanogenic proteins [32]. Furthermore, this selective inhibition of hydrogenotrophic methanogens forces a metabolic shift, redirecting metabolic hydrogen toward alternative sinks, a phenomenon substantiated by recent rumen-omics research [61,62,63].

Crucially, the reduced abundance of MCR subunit genes suggests a holistic suppression of the entire enzyme complex rather than isolated effects on individual subunits, mirroring the coordinated downregulation of methanogenic modules reported in other multi-omics studies [64,65]. It is worth noting that while the M-SEO group matched the methanogenesis-mitigating enzymatic profile of Agolin, the H-SEO group induced a slight decline in fermentation efficiency. This trade-off suggests that at high dosages, the broad-spectrum antimicrobial activity of SEO may extend beyond methanogens to affect other fibrolytic or fermentative bacteria [31].

One limitation of this in vitro study was the lack of an ethanol vehicle control. Although the final ethanol concentration was kept below 0.5% to minimize potential effects on the microbial community [21,22], future studies should include an ethanol vehicle control to better distinguish the effects of the essential oils from those of the solvent.

In summary, our results suggest that SEO may reduce CH_4_ emissions, possibly through effects on both the HDR system and the multi-component MCR complex. Such changes may disrupt electron flow and coenzyme regeneration [55,56], providing a possible molecular explanation for the methane-mitigating effects of essential oils observed in this and previous studies [57,66].

## 5. Conclusions

This in vitro study demonstrates that SEO can serve as an effective natural additive for mitigating CH_4_ emissions from dairy cows. A concentration of 200 mg/L (M-SEO) can reduce CH_4_ production by 17.5% while enhancing rumen fermentation through more total VFA production and increased microbial protein synthesis. These results reflect that SEO can selectively modulate the rumen microbiome, especially enriching the hydrogen-utilizing propionate producers, and suppressing the function of methanogenic enzymes and pathways. Future in vivo validation is warranted.

## Figures and Tables

**Table 1:** Ingredient and chemical composition of the basal diet.

Item ^1^	Basal Diet
Ingredient (% DM)	
Corn silage	28.42
Alfalfa hay	25.30
Corn	17.48
Oat grass	6.16
Barley	5.17
Cottonseed meal	4.06
DDGS	5.31
Soybean meal	5.26
Limestone	0.32
NaCl	0.31
CaHPO_4_	0.56
CaHCO_3_	0.36
Mineral premix ^2^	1.20
Chemical composition	
NE_L_ (MJ/kg) ^3^	6.29
CP (% DM)	15.02
Ether extract (% DM)	3.96
NDF (% DM)	41.11
ADF (% DM)	22.04
Ca (% DM)	0.82
P (% DM)	0.42

^1^ DM = dry matter; DDGS = Distillers dried grains with solubles; CP = Crude protein; NDF = Neutral detergent fiber; ADF = Acid detergent fiber. ^2^ Mineral premix, which provided the following per kg of premix: vitamin A 300,000 IU, vitamin D 385,000 IU, vitamin E 1455 IU, nicotinic acid amide 550 mg, Mn 930 mg, Zn 3600 mg, Fe 1200 mg, Cu 770 mg, Co 12 mg, Se 21 mg, I 50 mg. ^3^ NE_L_, estimated using the NRC (2001) [23] model.

**Table 2:** Effect of different doses of Spearmint essential oil and Agolin on gas production.

Item ^1^	Treatment ^2^					SEM	*p*-Value
	CON	L-SEO	M-SEO	H-SEO	AGL		
Total gas production, mL/g DM							
6 h	71.25	74.18	78.77	78.53	73.29	1.253	0.468
24 h	151.69 ^a^	150.71 ^a^	142.54 ^ab^	126.33 ^c^	134.37 ^bc^	4.055	<0.001
48 h	174.31	169.54	160.45	170.00	171.73	3.943	0.077
Methane, mL/g DM							
6 h	20.37	19.95	17.89	21.66	16.54	0.852	0.344
24 h	58.11 ^a^	54.99 ^ab^	47.93 ^bc^	46.58 ^c^	50.31 ^abc^	1.644	<0.001
48 h	72.55 ^a^	67.69 ^a^	59.35 ^b^	71.85 ^a^	70.19 ^a^	2.409	<0.001
Carbon dioxide, mL/g DM							
6 h	37.83	40.10	36.32	43.84	34.37	2.248	0.238
24 h	74.99 ^a^	74.33 ^a^	69.87 ^ab^	56.42 ^c^	59.52 ^bc^	3.180	<0.001
48 h	80.40	78.08	72.70	75.91	77.35	3.159	0.487
Hydrogen, mL/g DM							
6 h	0.47	0.41	0.46	0.47	0.30	0.020	0.180
24 h	0.77	0.76	0.90	0.77	0.76	0.063	0.426
48 h	0.89	0.90	1.01	0.91	0.88	0.070	0.507

^1^ DM = dry matter. ^2^ CON = without any additives; L-SEO, M-SEO, H-SEO = experimental diet plus 100, 200, and 400 mg/L spearmint essential oil, respectively; AGL = experimental diet plus 150 mg/L Agolin. ^a,b,c^ Values within a row with different superscripts differ significantly at *p* < 0.05.

**Table 3:** Effect of different doses of spearmint essential oil and Agolin on rumen fermentation parameters at 24 h.

Item ^1^	Treatment ^2^					SEM	*p*-Value
	CON	L-SEO	M-SEO	H-SEO	AGL		
pH ^†^	6.43 ^c^	6.44 ^bc^	6.45 ^abc^	6.46 ^ab^	6.47 ^a^	0.004	0.004
NH_3_-N, mg/dL	19.52 ^a^	19.35 ^a^	18.7 ^ab^	16.98 ^bc^	16.50 ^c^	1.138	<0.001
MCP, mg/dL	27.02 ^c^	31.14 ^bc^	35.05 ^ab^	38.12 ^a^	36.64 ^a^	1.700	<0.001
IVDMD ^†^, %	58.05	56.17	57.46	53.82	57.36	1.958	0.629
VFA, mmol/L							
Acetate	34.92	36.17	39.89	37.81	35.99	1.675	0.402
Propionate	7.13 ^b^	7.06 ^b^	9.21 ^a^	7.86 ^ab^	6.94 ^b^	0.386	0.014
Isobutyrate ^†^	0.47	0.41	0.79	0.45	0.54	0.063	0.064
Butyrate ^†^	4.79 ^b^	4.71 ^b^	6.33 ^a^	5.44 ^ab^	4.64 ^b^	0.301	0.032
Isovalerate ^†^	0.79 ^b^	0.8 ^b^	1.16 ^a^	0.93 ^ab^	0.75 ^b^	0.058	0.018
Valerate ^†^	0.25	0.24	0.43	0.34	0.31	0.061	0.088
Caproate ^†^	0.06 ^b^	0.12 ^ab^	0.29 ^a^	0.20 ^a^	0.06 ^b^	0.026	0.005
TVFA	48.41	49.52	58.1	53.04	49.23	1.968	0.042
A/P ratio	4.90	5.14	4.38	4.83	5.27	0.272	0.285

^1^ NH_3_-N = ammonia nitrogen; MCP = microbial crude protein; IVDMD = in vitro dry matter digestibility; TVFA = total volatile fatty acid; A/P ratio = ratio of acetate to propionate. ^2^ CON = without any additives; L-SEO, M-SEO, H-SEO = experimental diet plus 100, 200, and 400 mg/L spearmint essential oil, respectively; AGL = experimental diet plus 150 mg/L Agolin. ^a,b,c^ Values within a row with different superscripts differ significantly at *p* < 0.05. ^†^ Data were analyzed by the non-parametric Kruskal–Wallis test followed by Dunn’s post hoc test. All other variables were analyzed by one-way ANOVA followed by Tukey’s HSD test.

**Table 4:** Effect of different doses of spearmint essential oil and Agolin on alpha diversity of bacteria and archaea at 24 h.

Item	Treatment ^1^					SEM	*p*-Value
	CON	L-SEO	M-SEO	H-SEO	AGL		
Bacteria							
Shannon	8.47 ^b^	8.66 ^ab^	8.96 ^a^	8.86 ^ab^	8.32 ^b^	0.084	0.001
Simpson	0.985 ^bc^	0.987 ^ab^	0.989 ^a^	0.988 ^ab^	0.983 ^c^	0.0008	0.002
Pielou	0.567 ^bc^	0.579 ^ab^	0.598 ^a^	0.591 ^ab^	0.555 ^c^	0.0056	0.001
Chao1 ^†^	105.85	105.56	105.14	99.86	101.94	3.821	0.749
ACE	89.78	89.57	89.34	89.16	89.40	0.173	0.228
Archaea							
Shannon	8.077 ^b^	8.093 ^ab^	8.115 ^a^	8.096 ^ab^	8.078 ^b^	0.0076	0.035
Simpson ^†^	0.9875	0.9876	0.9879	0.9877	0.9876	<0.0001	0.094
Pielou	0.718	0.720	0.721	0.719	0.718	0.0007	0.062
Chao1	21.36	17.38	16.67	18.32	20.53	1.868	0.451
ACE ^†^	16.40	16.23	16.38	16.03	16.33	0.128	0.465

^1^ CON = without any additives; L-SEO, M-SEO, H-SEO = experimental diet plus 100, 200, and 400 mg/L spearmint essential oil, respectively; AGL = experimental diet plus 150 mg/L Agolin. ^a,b,c^ Values within a row with different superscripts differ significantly at *p* < 0.05. ^†^ Data were analyzed by the non-parametric Kruskal–Wallis test followed by Dunn’s post hoc test. All other variables were analyzed by one-way ANOVA followed by Tukey’s HSD test.

## Data Availability

The original contributions presented in this study are included in the article. Further inquiries can be directed to the corresponding author.
